# Cleft Palates—Cases in Practice

**Published:** 1895-12

**Authors:** Benjamin Simons


ARTICLE VIII.

### CLEFT PALATES—CASES IN PRACTICE.

BY DR. BENJAMIN SIMONS.

**Read before the South Carolina State Dental Society, July, 1895.**

*Gentlemen :*—I regret that I am unable to be with you and join in your deliberations. Business of a pressing nature compels my absence. I have not however forgotten our Association, and will tender, through the kindness of Dr. Smith, a few models and explanations, which I hope will serve to interest you.

The first case is a replacement of partial denture, jawbone and soft palate of a negro man, about forty years of age, affected with a tumor, which extended from the posterior border of the superior maxillary bone, on the right side, as far down as the lower part of the palato-pharyngeus muscle. The tumor has reached such a size as to make deglutition and speech very difficult, and would have resulted in death, but for the timely interference of Dr. Manning Simons, who placed the patient under chloroform and performed tracheotomy, an operation which it is needless to describe to you. He then made an incision in the median labial line, around the ala of the nose, and passing upward and outward about on a line with the lower border of the malar bone; then turned cheek down and removed, with incising forceps, the right side of the superior maxillary bone, with horizontal plate of palate, taking also greater part of the tensor, and levator palati muscles and paloto pharyngeus, leaving a portion of the palato glossus muscle. The patient was up and out in about four weeks after the operation.

I at first attempted to take an impression in modeling composition, which resulted in failure. I then saw that nothing but plaster could be used to advantage, this was dangerous but the only alternative, so I determined to try the experiment The patient was placed in the dental chair in upright position, my only assistant being an African boy. The plaster was made quite soft, I dipped same up with two mouth mirrors and placed in little by little just in bottom of V formed by the uncut remnant of the palato glossus muscle, at the same time pressing backward and behind the ragged edge of the palate, and so on carefully built up the root of the mouth. When set hard, removed pieces and trimmed to suit the curve of the mouth and palate. The piece was then varnished and oiled, and before replacing put some soft plaster under malar bone, and above the constriction of the ciccatrix, caused by the incision, then replaced piece, and when plaster had set, removed, which gave me a projection or outer wall of the antrum by which the right side of the jaw was supported. I then took plaster casts of the natural and artificial jaw in position; when set removed them, and took impression of the entire parts, taken seven or eight parts to do this. I then placed in articulator and made up as common plate with this exception, I had no flasks that would hold it, so took two of the White Company's wax boxes and made one and bound it tightly together with wire after carefully packing it, and vulcanized with excellent result. The patient's appearance was very much benefited, and his speech almost restored. I neglected to state that I hollowed out the palate more for the purpose of lightening the weight than forming the antrum, but it served the purpose of both.

The second is a lady of twenty-five, who has congenital cleft palate, with very imperfect speech. I saw her about two years ago. I put in one of Dr. Kingsley's soft rubber palates, the movements of which are dependent upon the motion of the remnants of the palate muscles as you well know. She wore this one year but with little or no success as to improvement of speech, though she could get a closure so as to be able to blow out a candle, so I determined to make her a hard rubber palate and try to educate the superior constrictor muscle, as it was useless to try longer with the palate muscles. So I made a plate bridging the hard palate and attached it to the teeth with clasps, to which plate was attached at the heel two pieces of metal curving down to about a quarter of an inch of touching the superior constrictor muscle and about on a line with the lower border of the velum. I covered this with wax in ball shape, but the wax would not conform to the shape properly, so I made a small ball of wax covered it with soft modelling composition and while warm replaced in mouth, and placed finger under ball to keep in position, and made patient swallow, and repeated same several times heating ball or composition each time until I got exactly the impress of the pharyngeal wall and remnants of palati muscles as shown in model sent. Then took the impression and run counter impressions of the piece in sections as described in first case. Removed model and put in a layer of rubber cutting a hole in bottom of first piece, then waxed up the balance filling same with soft plaster, thus making a ball within a ball, then opened and packed it as regular case, and vulcanized in same tin flask. Took plaster out of ball through hole cut in rubber as described, put in tin piece and covered with rubber and vulcanized. When finished found ball full of water, drilled hole and blew water out and filled with amalgam.

Plate was very light and serves the purpose tolerably well. Patient is improving in speech and has great comfort.

The third case was a negro girl about twenty-two years of age affected with non-malignant bony tumor of the inferior maxillary bone, size about four by three inches. Dr. R. Barnwell Rhett successfully removed jaw from first bicuspid on one side, to ramus on the other. Patient recovered in about four weeks with remarkably little disfiguration. When she came to me for replacement, the pterygoid muscles had pulled the remnant of the jaw within the arch, and I at first thought that I would have to put in an upper plate with guide so as to push out jaw for proper occlusion, but on second consideration resolved to try to cultivate the masseter and temporal muscles with artificial means, so instructed patient to pass finger in mouth and press jaw out, then close tightly. After one week to my surprise, I found that she could articulate the jaws without assistance, so pressed teeth open with rubber so as to get space for the clasps, she having only three teeth left in the lower part.

I then took impression of the remnant of the jaw and tissue with open mouth as usual, and proceeded to make the jaw. But on putting same in was surprised to find that the bite was entirely out, this led to investigation and I very soon found out that on opening the mouth, the muscles pulled up the tissue, which, when closed occupied a very different position, and that the only way to success was to take an impression when the mooth was shut. So I took an accurate impression with plaster of the remnant of the jaw and teeth, taking also the soft parts, as they were, then made a plate telescoping remnants and teeth accurately, put on clasps surrounding the bicuspid and half around the molar, with rubber on both sides of the jaw, also running a band approximating the curve of the jaw on the other side vulcanizing same, replaced in mouth and trimmed band so as not to press too heavily over on remnant of ramus on other side. I then removed piece and covered lower border of band replaced in mouth and made patient close and pressed lips in position, which gave me an impression of jaw correctly when closed. I then covered upper border with wax and set up teeth one by one until I secured an exact bite, and attached a convex rounded piece at extreme end of jaw so as to glide over and not catch under ramus as a rough end would do, and also to keep the jaw pressed outward, since the end having a different axis must slide to compensate for the shortage. Then removed piece and run counter dies in sections as described, opened same and removed the plaster and wax and replaced with rubber and vulcanized. Patient's appearance is tolerably well restored. Dr. Smith having seen the above cases will be able to give you a better idea of them.—*Southern Dental Journal and Luminary*.

